# KOH-activated micrometer-thick amorphous carbon nanofoam as a binder-free supercapacitor electrode with high-rate performance†

**DOI:** 10.1039/d5cc01916h

**Published:** 2025-07-14

**Authors:** Subrata Ghosh, Yifan Zhang, Giacomo Pagani, Raffaella Suriano, Marco Agozzino, Agnieszka Jastrzębska, Carlo S. Casari

**Affiliations:** a Micro and Nanostructured Materials Laboratory – NanoLab, Department of Energy, Politecnico di Milano via Lambruschini Milano 20156 Italy carlo.casari@polimi.it; b Faculty of Mechatronics, Warsaw University of Technology św. A. Boboli 8 Warsaw 02-525 Poland subrata.ghosh@pw.edu.pl; c Laboratory of Chemistry and Characterization of Innovative Polymers (ChIPLab), Department of Chemistry, Materials and Chemical Engineering “Giulio Natta”, Politecnico di Milano Piazza Leonardo da Vinci 32 20133 Milano Italy

## Abstract

We demonstrate an aqueous supercapacitor based on self-standing, porous, hydrophilic, and 26-μm-thick activated carbon nanofoam. The device delivers an areal (volumetric) capacitance of 95.4 mF/cm^2^ (18.3 F/cm^3^) at 2 mA with retention of 103% at 20 mA and achieves a voltage of 1 V, superior compared to pristine and annealed examples.

## Abstract 2

This study presents a simple route to prepare binder-free activated amorphous carbon nanofoam, which is explored as an electrochemical energy storage electrode.

## Graphical abstract

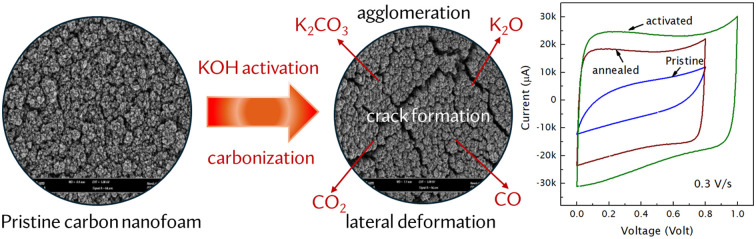

Carbon nanofoam—a 3D porous self-supporting structure comprising sp-, sp^2^- and sp^3^-hybridized carbons with the excellent characteristics of light weight, a volumetric void fraction greater than 90%, hydrophilicity in aqueous media, *etc.*—has received significant attention for use in various applications such as energy storage (supercapacitors and batteries),^1,2^ nuclear fusion,^3^ and laser-driven accelerators.^4^ However, as-grown or pristine hydrophilic carbon nanofoam delivers unsatisfactory supercapacitor performance. The specific capacitance of pristine and functionalized nanofoams falls into the range of a few mF cm^−2^ and the volumetric capacitance is around a few F cm^−3^.^1,5^ The supercapacitor performance of self-supporting structures, such as vertical graphene, was improved by post-processing activation with KOH^6^ or plasma,^7^ electrochemical processing for transformation into hydrophilic surfaces, or adding metal nanostructures to composites.^8,9^ In our previous study, we reported around a fifty-fold enhancement in the charge-storage performance of N-doped carbon nanofoam by annealing at a particular temperature.^10^ However, the annealed N-doped carbon nanofoam delivers an areal capacitance of 51.4 mF/cm^2^ and a volumetric capacitance of 4.7 F/cm^3^. Thus, there is still much space to enhance the supercapacitor performance further.

We solved the above-mentioned challenge by synthesizing self-standing porous carbon nanofoam using pulsed laser deposition, followed by KOH activation at a temperature of 750 °C for 2 hours under vacuum. To strengthen our findings, as-grown pristine nanofoam was annealed under vacuum with the same annealing conditions and without any KOH activation. A porous and columnar morphology is observed for the amorphous carbon nanofoam even after the annealing and activation processes (Fig. 1a–d; Fig. S1a–c and e–g, ESI†). The 3D porous morphology of the nanofoam assures higher accessibility and more interactions with electrolyte ions. Moreover, the porous carbon nanofoam was grown on the surface of a compact layer (Fig. 1c), which acts as an interface between the substrate and nanofoam and is effective for electron transportation to the external circuit. The change in annealed carbon nanofoam and activated carbon nanofoam compared to their pristine counterpart is observed in a thickness reduction from 35.6 μm to 33.4 μm and 26 μm, respectively (Fig. S1c and g, ESI†). The activated nanofoam shows cracks in the morphology (Fig. 1a–d) since the lateral deformation and agglomeration of nanofoam occurs once it is soaked in 6 M KOH solution.^11^ It has been reported that crack formation in a nanofoam takes place within the timescale of 1 s after the nanofoam is taken out from a liquid. The crack propagates when the nanofoam shrinks along with evaporation associated with the annealing and drying process. As a result, randomly shaped islands are observed, separated by deep channels. The average separation distance between the islands is estimated to be around 3 ± 1.8 mm. This common phenomenon, seen due to the liquid wetting and subsequent drying of nanofoam, is known as the *nanocarpet effect*.^11^

**Fig. 1:**
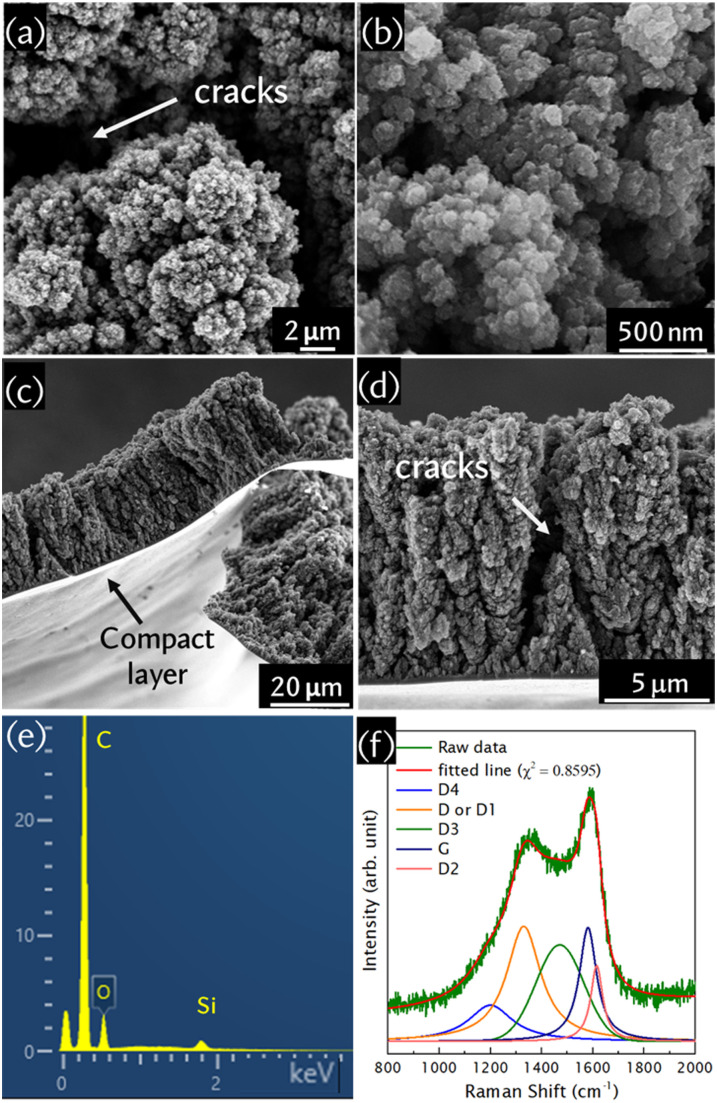
(a) and (b) Top view and (c) and (d) cross-sectional micrographs, (e) the energy dispersive X-ray spectrum, and (f) the Raman spectrum of the activated carbon nanofoam. The substrate used here is Si.

EDS analysis (Fig. S1d, ESI†) shows that pristine carbon nanofoam consists of carbon (87.3 at%), oxygen (3.4 at%), and nitrogen (9.3 at%). Since nitrogen was only used as a background gas during deposition to obtain a thick nanofoam^5^ and no oxygen gas was used during deposition, the presence of oxygen on the porous nanofoam surface originates from the physisorption of atmospheric oxygen once the material was taken out from the deposition chamber. Vacuum annealing at 750 °C increases the carbon content (95.9 at%) and lowers the amounts of functional groups (1.3 at% oxygen and 2.8 at% nitrogen) in the carbon nanofoam (Fig. S1h, ESI†). On the other hand, we have not seen any nitrogen in the activated nanofoam, which consists of 94 at% carbon and only 6 at% oxygen (Fig. 1e). Elemental mapping of the activated nanofoam is provided in Fig. S2a and b, ESI.† Therefore, soaking the pristine nanofoam in 6 M KOH introduces additional oxygen functional groups on the surface. During the vacuum annealing of KOH/carbon nanofoam, KOH reacts with carbon *via* the equation 6KOH + 2C → 2K + 3H_2_ + 2K_2_CO_3_, in which KOH is completely consumed at 600 °C, and the as-formed K_2_CO_3_ decomposes into CO_2_ and K_2_O at a temperature over 700 °C.^12^ In the annealed KOH/carbon nanofoam sample, the presence of potassium residue (2.1 at%) in the sample is observed from the EDX results (Fig. S3a–e, ESI†), and this was removed completely by washing several times followed by baking at 90 °C. Since the main products of KOH activation are H_2_, H_2_O, CO, CO_2_, K_2_O, and K_2_CO_3_, they react with nitrogen present in the nanofoam, producing volatile byproducts during activation. This result is in good agreement with the nanofoam thickness reduction upon activation (Fig. 1c and d). On the other hand, EDX spectra of pristine carbon nanofoam at the same accelerating voltage (10 keV) do not show any Si signature (Fig. S1d, ESI†), representing a densely packed nanofoam. However, the presence of a Si peak in the EDX spectrum (Fig. 1e) ensures that the activated nanofoam is more porous than its pristine counterpart. Elemental mapping of activated carbon nanofoam is provided in Fig. S2, ESI.† In addition, the morphology and elemental composition, with mapping, of activated carbon nanofoam directly grown on carbon paper are also supplied in Fig. S4, ESI.† Importantly, we noticed that the as-deposited nanofoam completely detached from the smooth Si substrate only once immersed in 6 M KOH, as aggressive KOH is a well-known chemical etchant that reacts with Si along the 〈100〉 direction preferentially, dissolving native oxides first; hence the nanofoam started to float (Fig. S5, ESI†). These results ensure that the film is freestanding and can be transferred easily to the desired substrate to be utilized for flexible energy storage applications. However, no detachment of the carbon nanofoam from the carbon paper substrate clearly suggests its excellent adhesivity with the carbon paper, as carbon is a constituent of both. We also deposited the nanofoam successfully on Ti, Au, and other substrates for different purposes, which indicates the versatility of pulsed laser deposition. Thus, activated carbon nanofoam can be prepared on flexible substrates, such as carbon cloth and thin metal foil, and hence it has potential for use in flexible electronics. In addition, it has already been reported that the electrical resistivity of this kind of nanoporous structure does not change upon bending, which is due to the damping of strain of the porous structure and compact layer.^13^

Raman spectra of the activated carbon nanofoam are shown in Fig. 1f and Fig. S6, ESI,† and it is fitted by a five-peak fitting model in Fig. 1f.^14^ The D-peak is a breathing vibration, and the G-peak is a stretching vibration of sp^2^ carbon. Furthermore, D4 is associated with the lattice vibrations of the sp^2^–sp^3^ bond, D3 is assigned to the amorphous structure, and D2 is related to the *E*_2g_ symmetry of the disordered graphitic structure.^14^ The full width at half maximum (FWHM) values of the D and G peaks are reduced upon annealing (FWHM of the D-peak: 152 cm^−1^; and the G-peak: 87 cm^−1^) compared with the pristine nanofoam (FWHM of the D-peak: 160 cm^−1^; and the G-peak: 88 cm^−1^), confirming the structural improvement. The blue shift of the G-peak from 1572 cm^−1^ to 1581 cm^−1^ indicates a higher content of nanocrystalline graphite in the amorphous carbon system, while the amount of fused aromatic rings and olefinic chain clusters is reduced.^15^ Functionalization also leads to an increase in the D-peak intensity and partial graphitization,^16^ which leads to the redshift of the D-peak. The reduced FWHM of the G-peak (82 cm^−1^) in the activated nanofoam is a clear indication of KOH-activation-induced structural changes. Moreover, we have seen changes in the D4 and D3 peaks, which were used to probe the oxygen content in graphene oxide.^17^ The shift in the D4 peak position from 1236 to 1200 cm^−1^ and the D3 peak position from 1465 to 1471 cm^−1^, and the increase in *I*_D3_/*I*_G_ from 0.75 to 0.85 and the D3 content from 2.7% to 27% in the activated nanofoam compared with the annealed nanofoam indicates the incorporation of oxygen into the structure. This result is in good agreement with the EDX results. Similar trends in peak position and FWHM values for the D-peak and G-peak are observed from the widely used two-peak fitting approach (Table S1, ESI†), but the five-peak fitting model is more informative for interpreting the structural changes in the carbon nanofoams. For further insights into oxygen incorporation in amorphous carbon, we performed static water contact angle measurements for all studied nanofoams (Fig. S7, ESI†). Unlike other porous carbon structures, which are hydrophobic, we found that water immediately spreads out once dropped, indicating a hydrophilic nature. The hydrophilic surface is highly desirable and very effective for efficient electrode/electrolyte interaction.^6–8^ The hydrophilicity could be due to the porous structure of the carbon nanofoam and functional groups attached on the surface.

Fig. S8a–c and e–g, ESI,† show cyclic voltammograms (CVs) at various scan rates and charge–discharge (CD) profiles at different currents, respectively, for all studied supercapacitors. Comparisons of CV and CD profiles for all devices are shown in Fig. 2a and b, respectively. The CD profiles are almost symmetric and near-linear for all the nanofoams. It is interesting to see that the activated nanofoam not only takes a longer time to discharge but also provides a higher stable device voltage of 1 V, and hence a higher energy density compared to the other nanofoam-based supercapacitors (Fig. 2c). The estimated areal (volumetric) capacitance values for the pristine, annealed, and activated nanofoam devices at a 2-mA discharge current (corresponding current density of 2.55 mA/cm^2^) are 52.6 (7.73), 102.2 (15.32), and 95.4 (18.33) mF/cm^−2^ (F/cm^3^), respectively (Fig. 2c and Fig. S8h, ESI†). Besides, impressive capacitance retention of 103% at a 20-mA discharge current is observed for the activated carbon nanofoam. This result is uncommon but seen in the reports on superwetted vertical graphene nanosheets, a nanoporous gold/MnO_2_ hybrid,^18^ N-doped hydrogenated amorphous carbon,^10^ carbon nanotubes,^19^ and a metal-free MXene.^20^ Post-test morphological inspections confirm the preservation of the porous and columnar morphology (Fig. S9a–c, ESI†) obtained in the activated carbon nanofoam before electrochemical testing (Fig. S4, ESI†). K^+^-ion intercalation in the structure is clearly observed, as a K content of 8 at% is obtained from EDX analysis (Fig. S9d–g, ESI†). Thus, although we performed post-test Raman analysis (Fig. S8h-I and Table S3, ESI†), a true investigation of changes in structure may not be appropriate to explain the excellent rate performance. To explain this similar phenomenon in our previous report^10^ on an aqueous symmetric supercapacitor made with N-doped hydrogenated amorphous carbon nanofoam vacuum-annealed at 600 °C, we performed *in situ* spectroelectrochemical measurements. We found a trend in the position and FWHM of the D- and G-peaks and a change in the D-to-G intensity ratio of the nanofoam with respect to scan rate.^10^ Thus, the excellent capacitance retention in the annealed and activated nanofoams obtained here is attributed to electrochemical activation process-induced structural changes during the charge–discharge process. The cyclic stability of the activated carbon nanofoam is also studied, as shown in Fig. 2d, and found to be better compared to its annealed counterpart. The cyclic stability can be improved further by minimizing the number of K^+^ ions trapped inside the nanofoam, which can be done by optimizing the porosity further by tuning the PLD process parameters and KOH activation process parameters. As can be seen from Fig. 2e, the Nyquist plot of the pristine nanofoam consists of a semicircle with a large radius of 4.9 Ω cm^2^ (also known as the charge-transfer resistance, obtained from equivalent electric circuit modelling; Table S5, ESI†) in the high-frequency region. Moreover, a higher pseudocapacitive contribution is obtained from the activated nanofoam compared to that from the annealed one (Table S5, ESI†), which is due to the higher amount of oxygen functional groups. On the other hand, a line almost parallel to the *Z*′′-axis without a significant semicircle is seen for the activated nanofoam, indicating its ideal supercapacitor behaviour. To probe ion diffusion into the structure, *Z*′ is plotted as a function of *ω*^−0.5^ (Fig. 2f). The smaller slopes for the annealed and activated nanofoam devices indicate better ionic diffusion into the porous nanofoam structure than the pristine counterpart. This can be attributed to the better structural quality of the modified nanofoams. Further, Bode plots of devices are shown in Fig. 2g. The estimated relaxation time constant at −45° and capacitor-resistor time constant at 120 Hz are 0.31 s and 0.36 s, respectively, demonstrating the ultrafast frequency response of the activated nanofoam-based supercapacitor.

**Fig. 2:**
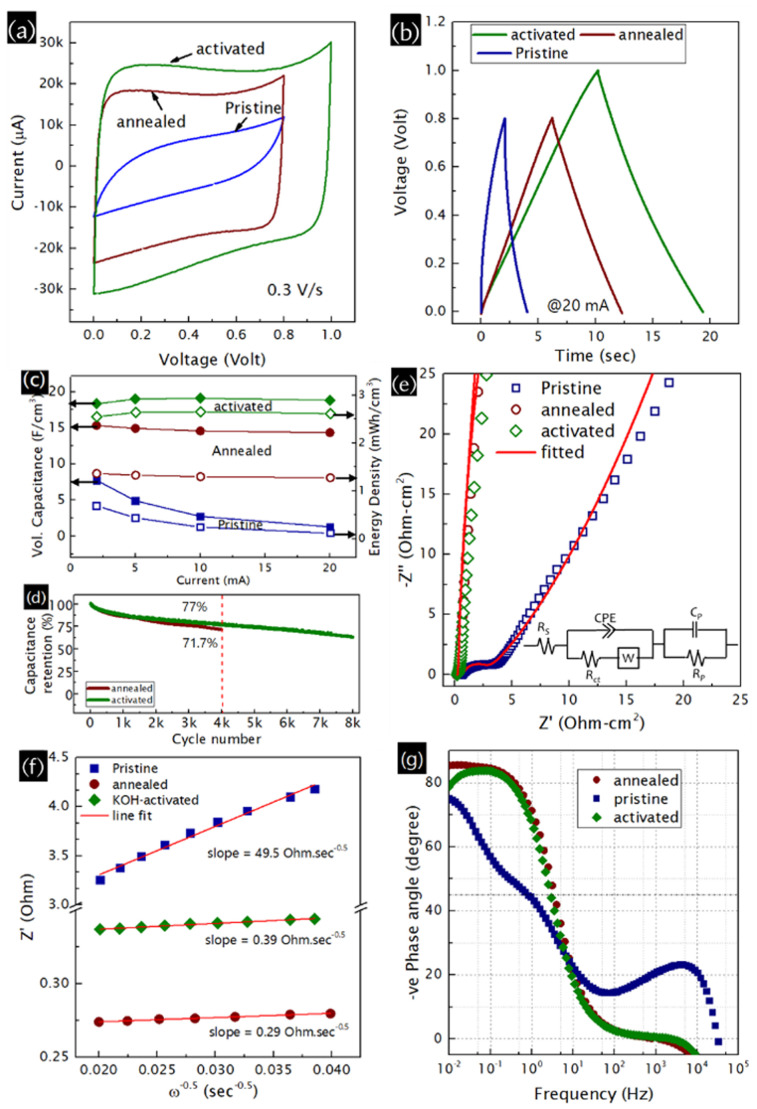
Comparisons of (a) cyclic voltammograms, (b) charge–discharge profiles, (c) volumetric capacitance and energy density at different currents, (d) cycling testing, (e) Nyquist plots, with the fitted equivalent electric circuit in the inset, (f) *Z*′ *vs. ω*^−0.5^ plots and (g) bode plots for the devices.

It is noteworthy to mention that we did not use any conductive additives and binders. The so-called *dead mass* of conductive additives and binders that is detrimental due to a lack of contribution to charge storage and the blocking of electrolyte ions from accessing the electrode surface. Thus, the binder-free porous activated nanofoam electrode could be promising for the fabrication of thin-film or micro- to macro-supercapacitors that are effective for portable and miniaturized on-chip microelectronic applications.^21–23^ The charge-storage performance of the activated nanofoam is compared with other binder-free supercapacitors, as shown in Table S4, ESI.† Further enhancement in the charge-storage performance of the nanofoam can be obtained by growing a thicker film by ablating the target for a longer time, by tuning the density of the nanofoam by allowing background gas along with nitrogen,^5^ by defect engineering during the pulsed laser deposition or post-treatment,^10^ and by tuning the activation process parameters (*e.g.* the activation medium and background gas). Moreover, we emphasize that the growth is reproducible,^10^ and it can be scaled further by translating the substrate holder in our set-up. However, synthesizing such binder-free, self-supporting, lightweight, and ultra-dense nanofoams is not feasible by approaches such as hydrothermal,^24^ sol–gel,^25^ chemical vapor deposition, and pyrolysis^26^ methods.

In summary, we proposed a strategy involving the KOH activation of pulsed-laser-deposited, binder-free, self-supporting, porous, and N-doped amorphous carbon nanofoam to enhance the charge-storage performance. The activation process leads to the nanocarpet effect, increases the porosity, improves the structural quality, and increases the sp^2^-carbon content with useful functional groups. In turn, the aqueous symmetric supercapacitor delivers an areal (volumetric) capacitance of 95.4 mF/cm^2^ (18.3 F/cm^3^) at 2.55 mA/cm^2^ with 103% retention at 25.5 mA/cm^2^ and excellent cycling stability and charge-storage kinetics. This demonstrates the potential of lightweight and thick nanofoams for portable and flexible supercapacitor applications.

S. G. performed the experiments, conceptualized and wrote the draft. R. S. did the wettability test. Y. Z. M. A. and G. P. helped with experiments and analyses. A. J. and C. S. C. assisted in conceptualization and edited the manuscript thoroughly.

S. G. and Y. Z. acknowledge Horizon Europe for the Marie Sklodowska-Curie postdoctoral Fellowship grant no. 101067998-ENHANCER and 101065920 – SCCAMC, respectively. C. S. C. acknowledges partial funding from the European Research Council (ERC) under the European Union's Horizon 2020 Research and Innovation Program ERC Consolidator Grant (ERC CoG2016 EspLORE Grant Agreement 724610, website: www.esplore.polimi.it), and funding by the project funded under the National Recovery and Resilience Plan (NRRP), Mission 4 Component 2 Investment 1.3 Call for Tender 1561 of 11.10.2022 of Ministero dell’Università e della Ricerca (MUR), funded by the European Union NextGenerationEU Award Project Code PE0000021, Concession Decree 1561 of 11.10.2022 adopted by Ministero dell’Università e della Ricerca (MUR), CUP D43C22003090001, Project “Network 4 Energy Sustainable Transition (NEST)”. A. J. acknowledges funding from Warsaw University of Technology within the Excellence Initiative: Research University (IDUB) programme.

## Conflicts of interest

There are no conflicts to declare.

## Supplementary Material

CC-061-D5CC01916H-s001

## Data Availability

All data are available in the manuscript and ESI.†
