# Supplementary material for: KOH-activated micrometer-thick amorphous carbon nanofoam as a binder-free supercapacitor electrode with high-rate performance
Source: Chem Commun (Camb). 2025 Jul 14;61(68):12797–800. doi: 10.1039/d5cc01916h (PMC12305501; doi:10.1039/d5cc01916h)
Supplement: CC-061-D5CC01916H-s001 [file CC-061-D5CC01916H-s001.pdf]

Supplementary Information (SI) for ChemComm.
This journal is © The Royal Society of Chemistry 2025

ChemComm

SUPPLIMENTARY INFORMATION

DOI: 10.1039/x0xx00000x

KOH-activated micrometer-thick amorphous Carbon Nanofoam as binder-free Supercapacitor electrode with high-rate performance

Supplementary Information contains methodology and experimental details, additional scanning electron micrographs, energy-dispersive X-ray spectra, and post-mortem characterizations of carbon nanofoams. Cyclic voltammogram at different scan rates, charge-discharge profile at different currents, plot of areal capacitance and additional results of all studied nanofoam are also provided. A comparison table on the supercapacitor performance of activated carbon nanofoams with the binder-free thin film supercapacitors reported in existing literatures, and table on fitting parameters and extracted parameters are also provided.

Synthesis methodology

Pristine amorphous carbon nanofoams were prepared by pulsed laser deposition (PLD) at room temperature using graphite (2-inch diameter) target with a purity of 99.99% (purchased from Testbourne B. V.). The *ns*-PLD setup exploits the second harmonic (λ = 532 nm) of a Q-switched Nd:YAG laser (pulse duration of 5–7 ns), with a repetition rate of 10 Hz and a fluence of 6.5 J/cm^2. The graphite target was placed on a rotating/translating holder to ablate the target uniformly, while the Si (100) and carbon paper substrates were placed on the rotating substrate holder to deposit the nanostructure homogeneously over the desired area. The target-substrate separation was fixed as 40 mm. Prior to the deposition, the chamber was evacuated down to the base pressure of 2×10^{-3} Pa using first a rotary followed by a turbomolecular pump. The deposition was carried out under 99% N_2 gas for 2 min at the pressure of 5 Pa followed by 30 min at the pressure of 300 Pa. The deposition unit was vented, and the as-deposited nanostructures, named as pristine, were taken out for further treatment and to investigate the morphology, structure, and electrochemical properties as supercapacitor electrodes.

For the annealed nanofoams, as-grown foams were placed inside the tubular furnace, annealed at 750 °C for 2 hrs under vacuum at the pressure around 10^{-3} Pa, and cooled down naturally to room temperature. The heating rate for the annealing was 4 °C/min. To prepare the KOH-activated nanofoam, as-grown nanofoam were dipped inside 6M KOH for 5 hrs and then loaded into the tubular furnace and the similar annealing process was carried out. After removing it from the furnace, the sample were washed thoroughly by deionized water until pH of the solution reached around 6. Thereafter, the washed samples were dried in the oven overnight at the temperature of 90 °C.

Microscopy and Spectroscopy.

The morphological investigation of all nanofoams were carried out by field-emission scanning electron microscope (FESEM, ZEISS SUPRA 40, Jena, Germany). Energy Dispersive X-ray (EDX) spectroscopy was employed at the acceleration voltage of 5 kV to evaluate the local chemical composition, using a Peltier-cooled silicon drift detector (Oxford Instruments) and the Aztec software for quantification.
Raman spectra of all samples were recorded using Renishaw *InVia* Raman spectrometer, where each Raman spectrum was recorded using the 514.5 nm excitation radiation from an Ar^+ laser source with a power of 0.4 mW, an 1800 line/mm grating spectrometer and a 50× objective lens, with 20 accumulations for 10 s each. Among the models to fit the first-order Raman spectrum of carbon structures, we adopted two-peaks fitting model - Lorentzian D-peak and Breit-Wigner-Fano lineshaped G-peak fitting (Fig. S5a, SI†), and five-peaks fitting model (Fig. 1f, S5b-c, SI†)). Former one is mostly used and proposed by Ferrari et al.,[1] whereas the later one is anticipated to establish the structure-property relationship better.[2] Fitting details and extracted parameters after fitting are supplied in table S1-2 of SI†, respectively.

 This journal is © The Royal Society of Chemistry 20xx

Wettability measurement.

Water contact angle measurements for all carbon nanofoam samples were carried out at room temperature using an OCA 15plus instrument (Dataphysics Co., Filderstadt, Germany), equipped with a CCD camera to capture side views of drop images, and a 500 µL Hamilton syringe to dispense water droplets. Water for chromatography (LC-MS Grade, LiChrosolv®) supplied by Merck (KGaA, Darmstadt, Germany) was used as a probe liquid. The dispensed volume for static measurements was 1 µl.

Electrochemical measurements.

The electrochemical performances of the nanofoams were investigated in a 2-electrode configuration using Swagelok Cell (SKU: ANR-B01, Singapore), basic 6M KOH (ACS reagent, sigma-aldrich, ≥85%) used as the aqueous electrolyte. The cell was assembled by sandwiching modified separator-soaked-electrolytes between two symmetric carbon nanofoams grown directly on carbon paper. Prior to the test, electrodes and modified separator were dipped into the electrolyte solution for 1hr, and the cyclic voltammetry was conducted within the electrochemical stable voltage range at 10 mV/s for 20 cycles. Charge-discharge tests at different currents from 2 to 20 mA were recorded using a palmsens4 electrochemical workstation (PALMSENS, The Netherland). The areal capacitance of device is calculated using the equation: of $C_{areal} = \frac{I_d \cdot \int V(t).dt}{A.\int V.dV}$, where, I_d is the discharge current, *A* is the geometric area of the electrode, t is the charging/discharging time and *V* is the voltage of the device. The volumetric capacitance of device obtained by dividing areal capacitance by total thickness of both electrodes. The relaxation time constant is calculated from the impedance spectra at 120 Hz using the equation: $\tau_{RC} = {-Z'}/{2\pi f Z''}$, where Z′ and Z″ are the real and imaginary components of impedance. Impedance spectra were fitted by ZSim software Demo version.

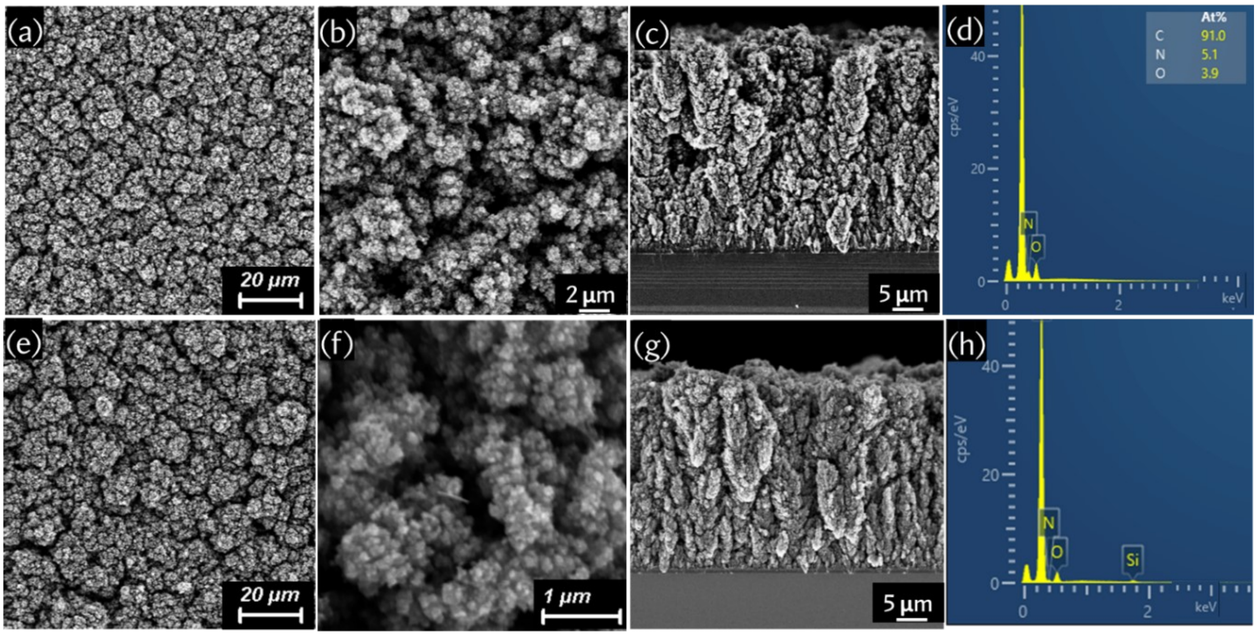

Fig. S1: Scanning electron micrograph of pristine pulsed laser-deposited amorphous carbon nanofoam. (a-b) top-view with different magnifications and (c) cross-sectional view. (d) Energy dispersive X-ray spectra. Scanning electron micrograph of vacuum annealed amorphous carbon nanofoam. (e-f) top-view with different magnifications and (g) cross-sectional view. (h) Energy dispersive X-ray spectra.

This journal is © The Royal Society of Chemistry 20xx

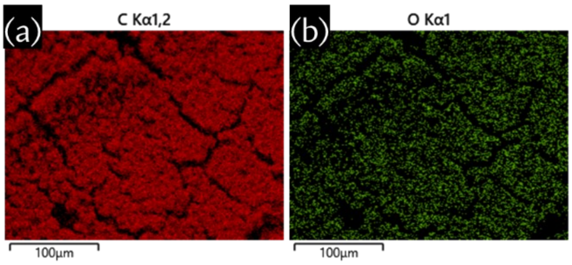

Fig S2: Elemental mapping of activated carbon nanofoam grown on Si. (a) C $K_{\alpha}^{1,2}$ and (b) O K_{α}^{1}.

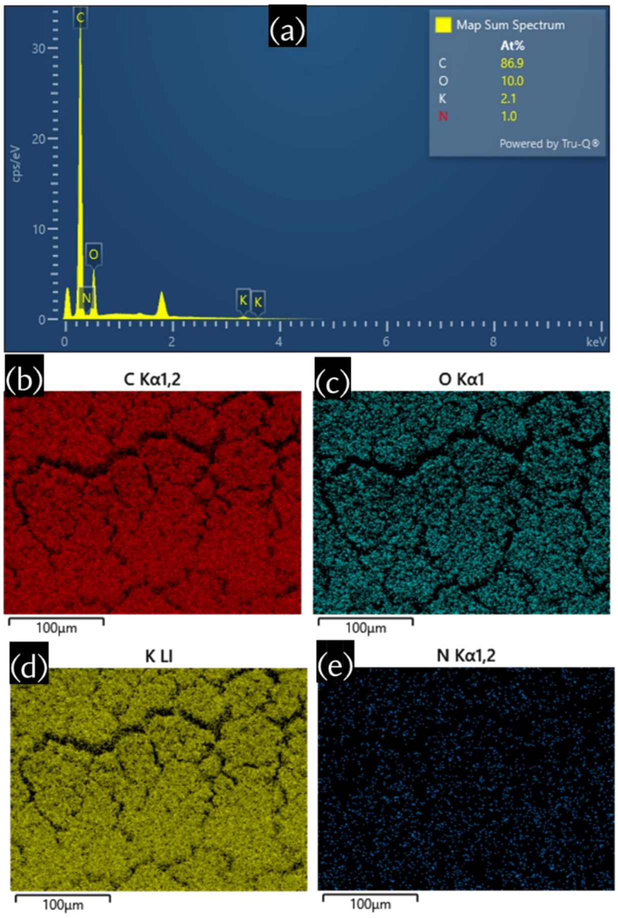

Fig S3: (a) Energy dispersive X-ray spectra of activated nanofoam before washing with corresponding elemental mapping for (a) carbon, (b) oxygen, (c) potassium, and (d) nitrogen.

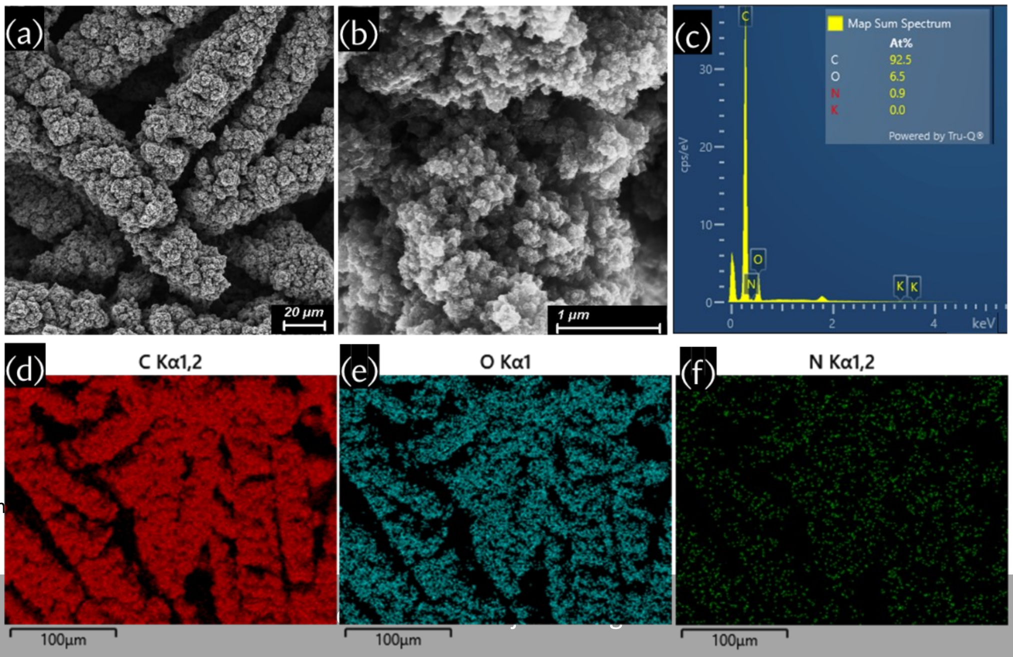

Fig S4: (a-b) scanning electron micrograph at different magnifications, and (c) Energy-dispersive X-ray spectra of activated carbon nanofoam grown on carbon paper. Corresponding elemental mapping- (d) C K_{α}[1,2], (e) O K_{α}[1] and (f) N K_{α}[1,2]

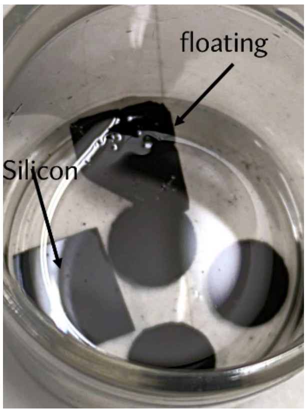

Fig. S5: Photograph of immersed pristine carbon nanofoams in 6M KOH solution. Foam grown on Silicon substrate is started to float after certain time of immersion, whereas other three circular discs are carbon foam coated carbon papers.

Table S1: Details of fitting of first order Raman spectra of carbon nanofoams

Five peaks fitting using WIRE 3.2 software			Two peaks fitting using ORIGIN software	
Peaks	Limit in peak position (cm^{-1})	lineshape	Peaks	lineshape
D4	[1050-1300]	Lorentzian		
D1 or D	[1300-1400]	Lorentzian	D	Lorentzian
D3	[1400-1550]	Gaussian		
G	[1550-1600]	Lorentzian	G	Breit-Wigner-Fano
D2	[1590-1630]	Lorentzian		

Table S2: Spectral parameters for the Raman bands of pristine, annealed and KOH-activated samples. Laser wavelength used is 514.5 nm. (Pos.: position and Γ: FWHM) The D3-content defined as (area of D4-peak $\times$100) / (sum of area of all five peaks) %

Five-peak fitting result [3,2]															
**Peaks**	**D4**			I_{D4}/I_G	**D**		**D3**			I_{D3}/I_G	**G**			**D2**	
	Pos. (cm^{-1})	Γ (cm^{-1})	content (%)		Pos. (cm^{-1})	Γ (cm^{-1})	Pos. (cm^{-1})	Γ (cm^{-1})	content (%)		Pos. (cm^{-1})	Γ (cm^{-1})	I_D/I_G (A_D/A_G)	Pos. (cm^{-1})	Γ (cm^{-1})
Pristine	1253	183	15	0.33	1363	160	1495	146	13	0.53	1576	88	0.97 (1.77)	1617	71
annealed	1236	186	1.3	0.33	1345	152	1465	249	2.7	0.75	1572	87	1.00 (1.75)	1610	48
activated	1200	209	1.4	0.32	1330	162	1471	221	27	0.85	1581	82	1.01 (2.00)	1615	57
Two-peak fitting result															

This journal is © The Royal Society of Chemistry 20xx

Pristine					1368	279					1585	126	0.75 (1.44)		
annealed					1366	268					1585	115	0.93(1.86)		
activated					1357	231					1591	104	0.80 (1.54)		

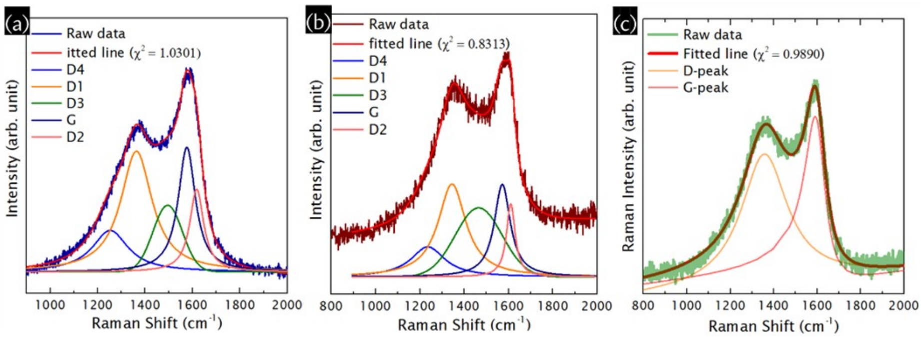

Figure S6: Raman spectra of (a) pristine carbon nanofoam fitted with five peaks fitting model, (b) annealed carbon nanofoam fitted with five peaks fitting model, and (c) activated carbon nanofoam fitted with two peaks fitting model.

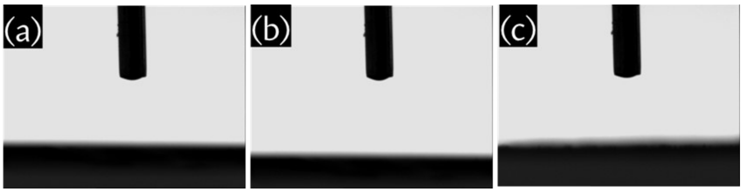

Figure S7: Water contact angle of (a) pristine, (b) annealed and (c) activated amorphous carbon nanofoams.

Please do not adjust margins

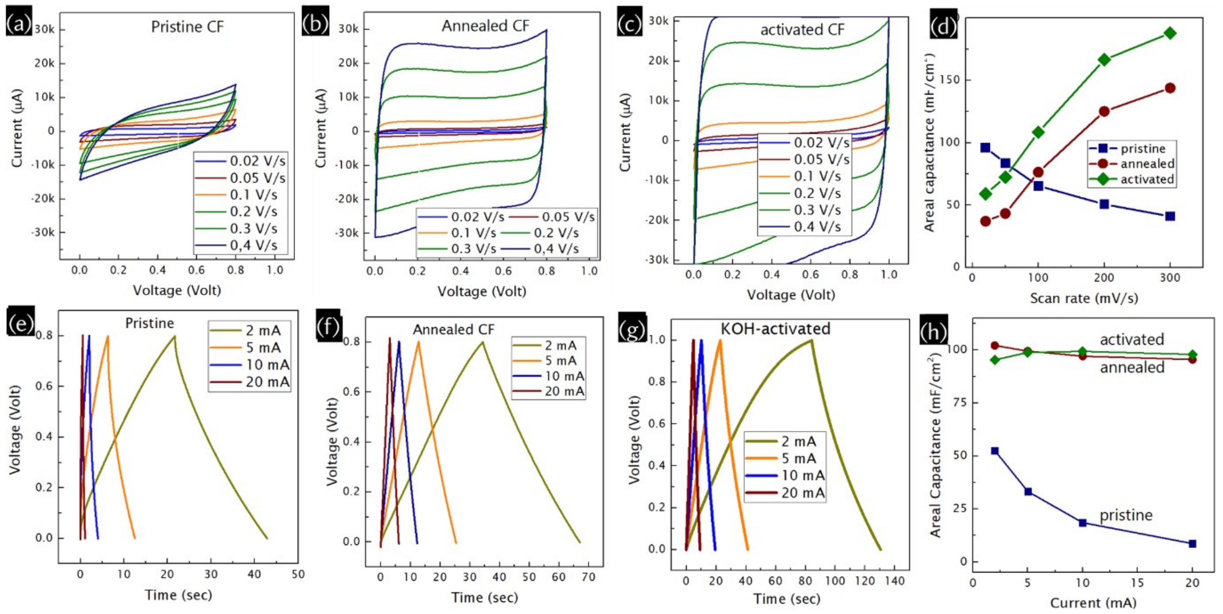

Fig. S8: Cyclic voltammogram of symmetric supercapacitors at different scan rates of (a) as-grown, (b) annealed, and (c) activated amorphous carbon nanofoam. (d) Areal capacitance of all supercapacitors as a function of scan rate. Charge-discharge profile of symmetric supercapacitors at different currents of (e) as-grown, (f) annealed, and (g) activated amorphous carbon nanofoam. (h) Areal capacitance of all carbon nanofoams as a function of charge/discharge current.

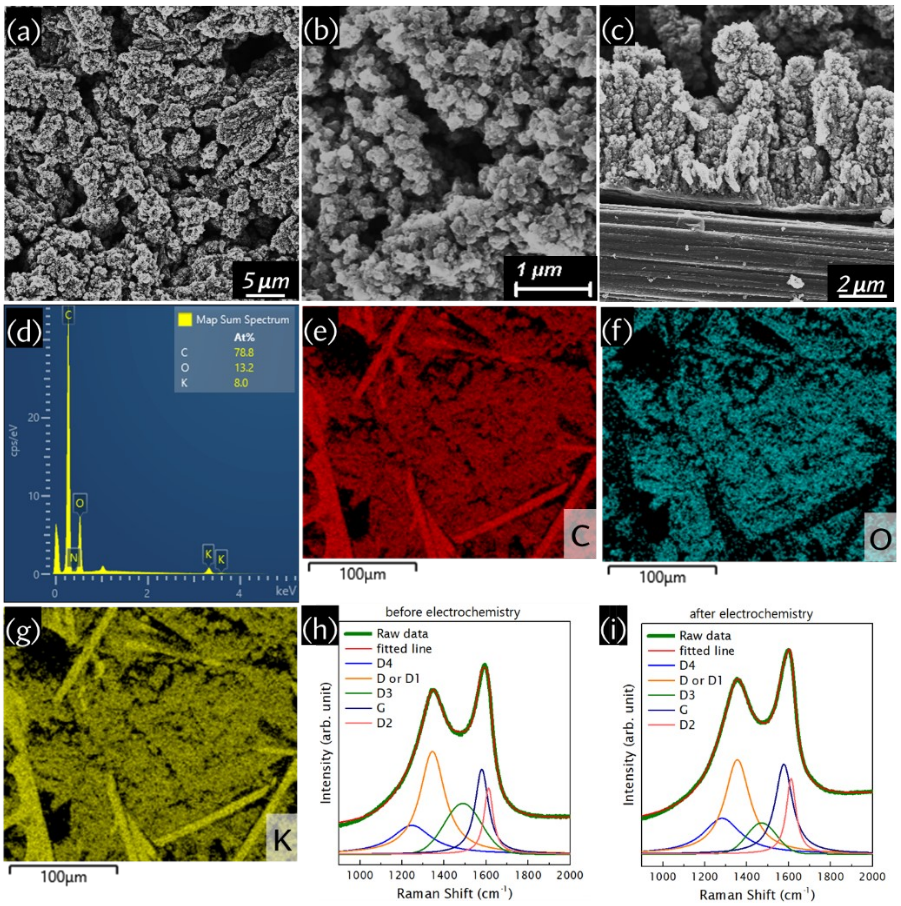

Fig. S9: Post-mortem analysis of activated carbon nanofoams on carbon paper substrate. (a-c) scanning electron micrograph at different magnifications, and (d) Energy-dispersive X-ray spectra and corresponding elemental mapping- (e) C $K_\alpha^{1,2}$, (f) O K_α^{1} and (g) N $K_\alpha^{1,2}$ Raman spectra (h) before and (i) after electrochemical measurement.

Table S3: Spectral parameters for the Raman bands of activated carbon nanofoams on carbon paper.(Pos.: position and Γ: FWHM)

Peaks	D4			I_{D4}/I_G	D		D3				G			D2	
	Pos. (cm^{-1})	Γ (cm^{-1})	content (%)		Pos. (cm^{-1})	Γ (cm^{-1})	Pos. (cm^{-1})	Γ (cm^{-1})	content (%)	I_{D3}/I_G	Pos. (cm^{-1})	Γ (cm^{-1})	I_D/I_G, A_D/A_G	Pos. (cm^{-1})	Γ (cm^{-1})
before	1247 (±10)	224 (±12)	17.2 (±0.5)	0.38 (±0.05)	1345 (±2)	160 (±14)	1491 (±1)	217 (±10)	19.4 (±1.1)	0.64 (±0.04)	1578 (±2)	80 (±3)	1.18 (±0.03), 2.34 (±0.08)	1612 (±1)	52 (±2)
after	1276 (±8)	219 (±30)	19.1 (±1.0)	0.37 (±0.03)	1356 (±4)	145 (±2)	1485(±14)	163 (±10)	9.4 (±1.3)	0.36 (±0.05)	1579 (±2)	94 (±6)	1.15 (±0.15), 1.79 (±0.31)	1612 (±1)	55 (±3)

Table S4: Supercapacitor performance of binder-free electrode ("//" stands for asymmetric supercapacitor)

Electrode and electrolyte	Thickness	Areal capacitance, Vol. capacitance	Rate performance	Voltage, electrolyte used	Cycle stability
**Result of device**					
Activated carbon nanofoam– This work	26 µm	95.4 mF/cm^2, 18 F/cm^3 at 2.55 mA/cm^2	103% at 25.5 mA/cm^2	1V, 6M KOH/modified propylene membrane	63.5% after 8000 cycles at 25.5 mA/cm^2
Annealed hydrogenated N-doped carbon nanofoam[4]	~ 57 µm	37 mF/cm^2 or 3.2 F/cm^3 at 1.27 mA/cm^2	88.5% at 12.7mA/cm^2	1V, 6M KOH/modified propylene membrane	87% after 10000 cycles at 2.55 mA/cm^2
Etched porous carbon skeleton/wood-derived carbon [5]	500 µm	1750 mF/cm^2, 17.5* F/cm^3 at 5 mA/cm^2	58 % at 30 mA/cm^2	1 V, polyvinyl alcohol-H_3PO_4 gel electrolyte	98.5 % after 20000 cycles at mA/cm^2
wood-derived carbon [6]	500 µm	2650 mF/cm^2, 26.5* F/cm^3 at 5 mA/cm^2	55.5 % at at 30 mA/cm^2	1 V, polyvinyl alcohol-H_3PO_4 gel electrolyte	97.3 % after 20000 cycles at mA/cm^2
superwetted vertical graphene nanosheets [7]	415 nm	2 mF/cm^2 at 100 mV/s	99.3 % at 800 mV/s	0.8 V, 1 M KOH gel electrolyte	80 % after 5000 cycles at 0.1 mA/cm^2
cumulenic *sp*-carbon atomic wires wrapped polymer [8]	40 µm	2.4 mF/cm^2 at 20 mV/s	54 % at 1 V/s	1 V, 6 M KOH/modified propylene membrane	85 % after 10000 cycles at 0.1 mA
		2 mF/cm^2 at 20 mV/s	36 % at 1 V/s	1 V, 1 M Na_2SO_4/modified propylene membrane	54 % after 10000 cycles at 0.1 mA
TiO_xN_{1-x} nanogrid [9]	-	2.6 mF/cm^2 at 10 mA/cm^2	-	0.8 V, 1 M KCl	88.9 % after 10000 cycles
	-	0.73 mF/cm^2 at 10 mA/cm^2	-	2.5 V, TEA-BF4/AN	83.9 % after 10000 cycles
	-	0.95 mF/cm^2 at 10 mA/cm^2	-	EMIM-TFSI	66.2 % after 10000 cycles
SiC nanowires@carbon nanotubes hybrid conductive network [10]	-	34.57 mF/cm^2 at 0.2 mA/cm^2	62.68 % at mA/cm^2	1 V, 2M KCl	107.1 % after 10000 cycles at 10 mA
MoS_2//V_2O_5 (asymmetric supercapacitor) [11]	-	11 mF/cm^2 at 0.8 mA	36.16* % at 2 mA	1.4 V, 1 M Na_2SO_4/filter paper	80 % after 5000 cycles at 3.5 mA
MnO_2 coated vertical graphene//Fe_3O_4 coated vertical graphene[12]	-	76 mF/cm^2 at 2.5 mA/cm^2	50 % at 10 mA	2.6 V, 1M $NaClO_4$/filter paper	79 % after 12000 cycles
Bismuth ferrite/graphene composite[13]	-	9 mF/cm^2 at 10 mV/s	32.4* % at 1 V/s	0.9 V, 1 M Na_2SO_4	95% after 5000 cycles
**Result of single electrode**					
Activated carbon nanofoam– This work	26 µm	381.6# mF/cm^2, 72 F/cm^3 at 2.55 mA/cm^2	103% at 25.5 mA/cm^2	1V, 6M KOH/modified propylene membrane	-
boron-doped ultranano- crystalline diamond[14]	140-185 nm	0.0784 mF/cm^2 at 20 mV/s	16.3* % at 100 mV/s	1V vs Ag/AgCl (3M KCl), 1 M Na_2SO_4	80% after 2000 cycles
boron-doped micro-crystalline diamond[14]	150 nm	0.0852 mF/cm^2 at 20 mV/s	18.5* % at 100 mV/s		
MnO_2 coated vertical graphene[12]	-	118 mF/cm^2 at 10 mV/s	29.66* % at 200 mV/s	1.4 V *vs* Ag/AgCl (3M KCl), 1M $NaClO_4$	Stable upto 2000 cycles
Fe_2O_3 coated vertical graphene[12]	-	151.11 mF/cm^2 at 10 mV/s	28.24* % at 200 mV/s	1.35 V *vs* Ag/AgCl (3M KCl), 1M $NaClO_4$	75.7 % after 2000 cycles
ZnO/Carbon nanowalls shell/core nanostructures[15]	800 nm	4.2 mF/cm^2 at 40 µA/cm^2	71.43* % at 200 µA/cm^2	0.7 V *vs* Ag/AgCl (3M KCl), 1 M KCl	Around 300% after 26000 cycles
TiO_XN_Y[16]	-	62 mF/cm^2 at 1 mA/cm^2	17.4*% at 10 mA/cm^2	1.1 V vs Hg/HgO, 1M KOH	100% after 1500 cycles
TiN[17]	-	26.9 mF/cm^2 at 1 mA/cm^2	60.8* % at 5 mA/cm^2	0.8 V vs Ag/AgCl (3M KCl), 0.5 M H_2SO_4	-
NbN[17]	-	39.6 mF/cm^2 at 1 mA/cm^2	62.5* % at 5 mA/cm^2		-
TiNbN[17]	-	59.3 mF/cm^2 at 1 mA/cm^2	65.8% at 10 mA/cm^2		94.2% at 5 mA/cm^2 after 20000 cycles

* For the volumetric capacitance, one needs to divide the areal capacitance of device by two times of thickness of each electrode.[18] Rate performance is estimated from the figure provided in cited reference using the WebPlotDigitizer software. #: calculated single electrode capacitance =4x device capacitance.

Please do not adjust margins

Table S5: Extracted equivalent electrical circuit parameters of fitted Nyquist plot for all carbon nanofoams.

Circuit parameters	Pristine carbon nanofoam	Annealed carbon nanofoam	Activated carbon nanofoam
Equivalent series resistance (R_S) in Ω-cm^2	0.7858	0.001321	0.01172
Constant Phase Element (*CPE*) in S-sec^n/cm^2	0.01552	0.1357	0.1439
n ($0<n<1$)	0.4278	0.9691	0.9643
Charge-transfer resistance (R_{ct}) in Ω-cm^2	4.932	0.03795	0.09981
Warburg resistance (*W*) in S-sec$^{0.5}$/cm^2	0.0336	0.000532	0.002537
Pseudocapacitance (C_P) in F/cm^2	0.07711	4.934E-8	1.045E-6
Surface resistance related to C_P (R_P) in Ω-cm^2	4217	0.1838	0.2103

References

1 A. C. Ferrari and J. Robertson, *Phys. Rev. B*, 2000, **61**, 14095–14107, DOI:10.1103/PhysRevB.61.14095.

2 Z. E. Brubaker, J. J. Langford, R. J. Kapsimalis and J. L. Niedziela, *J. Mater. Sci.*, 2021, **56**, 15087–15121, DOI:10.1007/s10853-021-06225-1.

3 A. Sadezky, H. Muckenhuber, H. Grothe, R. Niessner and U. Pöschl, *Carbon N. Y.*, 2005, **43**, 1731–1742, DOI:10.1016/j.carbon.2005.02.018.

4 S. Ghosh, G. Pagani, A. Macrelli, A. Calloni, G. Bussetti, A. Lucotti, M. Tommasini, R. Suriano, V. Russo, A. M. Jastrzkebska, C. Casiraghi, A. L. Bassi and C. S. Casari, *arXiv:2503.13264*.

5 J. Ouyang, X. Wang, L. Wang, W. Xiong, M. Li, Z. Hua, L. Zhao, C. Zhou, X. Liu, H. Chen and Y. Luo, *Carbon N. Y.*, 2022, **196**, 532–539, DOI:10.1016/j.carbon.2022.05.011.

6 J. Ouyang, M. Zhang, W. Xiong, L. Zhou, L. Zhao, Z. Li, C. Zhou, H. Chen, Y. Luo, S. Fang and R. H. Baughman, *J. Colloid Interface Sci.*, 2024, **671**, 145–153, DOI:10.1016/j.jcis.2024.05.172.

7 G. Sahoo, S. R. Polaki, S. Ghosh, N. G. Krishna and M. Kamruddin, *J. Power Sources*, 2018, **401**, 37–48, DOI:10.1016/j.jpowsour.2018.08.071.

8 S. Ghosh, M. Righi, S. Melesi, Y. Qiu, R. R. Tykwinski and C. S. Casari, *Carbon N. Y.*, 2025, **234**, 119952, DOI:10.1016/j.carbon.2024.119952.

9 Z. Wang, Z. Li and Z. Zou, *J. Power Sources*, 2015, **296**, 53–63, DOI:10.1016/j.jpowsour.2015.07.040.

10 H. Liu, X. Zhang, K. Li, Q. Cui, L. Han, Q. Shen, H. Li and X. Yin, *Carbon N. Y.*, 2024, **228**, 119411, DOI:10.1016/j.carbon.2024.119411.

11 A. Pullanchiyodan, G. T. Haridasan, P. Sreeram, A. Das, N. T. M. Balakrishnan, P. Raghavan and A. C. Hegde, *Energy & Fuels*, 2024, **38**, 3445–3457, DOI:10.1021/acs.energyfuels.3c03429.

12 S. Ghosh, S. R. Polaki, G. Sahoo, E.-M. Jin, M. Kamruddin, J. S. Cho and S. M. Jeong, *J. Ind. Eng. Chem.*, 2019, **72**, 107–116, DOI:10.1016/j.jiec.2018.12.008.

13 A. Soam, R. Kumar, M. C, M. Singh, D. Thatoi and R. O. Dusane, *J. Alloys Compd.*, 2020, **813**, 152145, DOI:10.1016/j.jallcom.2019.152145.

14 S. Suman, D. K. Sharma, O. Szabo, B. Rakesh, M. Marton, M. Vojs, A. Vincze, S. P. Dutta, U. Balaji, D. Debasish, R. Sakthivel, K. J. Sankaran and A. Kromka, *J. Mater. Chem. A*, 2024, **12**, 21134–21147, DOI:10.1039/D3TA07728D.

15 A. Guerra, A. Achour, S. Vizireanu, G. Dinescu, S. Messaci, T. Hadjersi, R. Boukherroub, Y. Coffinier and J.-J. Pireaux, *Appl. Surf. Sci.*, 2019, **481**, 926–932, DOI:10.1016/j.apsusc.2019.03.204.

16 A. Ramadoss, N. Swain, G. Saravanan, S. Z. Noby, K. Kirubavathi, L. Schmidt-Mende and K. Selvaraju, *J. Mater. Sci. Mater. Electron.*, 2022, **33**, 9669–9678, DOI:10.1007/s10854-021-07644-1.

17 B. Wei, F. Ming, H. Liang, Z. Qi, W. Hu and Z. Wang, *J. Power Sources*, 2021, **481**, 228842, DOI:10.1016/j.jpowsour.2020.228842.

18 J. Xie, P. Yang, Y. Wang, T. Qi, Y. Lei and C. M. Li, *J. Power Sources*, 2018, **401**, 213–223, DOI:10.1016/j.jpowsour.2018.08.090.

 This journal is © The Royal Society of Chemistry 20xx
